# A Review of Authenticity and Authentication of Commercial Ginseng Herbal Medicines and Food Supplements

**DOI:** 10.3389/fphar.2020.612071

**Published:** 2021-01-11

**Authors:** Mihael Cristin Ichim, Hugo J. de Boer

**Affiliations:** ^1^ “Stejarul” Research Centre for Biological Sciences, National Institute of Research and Development for Biological Sciences, Piatra Neamt, Romania; ^2^ Natural History Museum, University of Oslo, Oslo, Norway

**Keywords:** ginseng, Panax, identification, adulteration, contamination, herbal product, herbal medicine, food supplement

## Abstract

Ginseng traditional medicines and food supplements are the globally top selling herbal products. *Panax ginseng*, *Panax quinquefolius* and *Panax notoginseng* are the main commercial ginseng species in herbal medicine. Prices of ginseng products vary widely based on the species, quality, and purity of the used ginseng, and this provides a strong driver for intentional adulteration. Our systematic literature search has reviewed the authenticity results of 507 ginseng-containing commercial herbal products sold in 12 countries scattered across six continents. The analysis of the botanical and chemical identity of all these products shows that 76% are authentic while 24% were reported as adulterated. The number of commercial products as well as the percentage of adulteration varies significantly between continents, being highest in South America (100%) and Australia (75%), and lower in Europe (35%), North America (23%), Asia (21%) and Africa (0%). At a national level, from the five countries for which more than 10 products have been successfully authenticated, the highest percentage of adulterated ginseng products were purchased from Taiwan (49%), followed by Italy (37%), China (21%), and USA (12%), while all products bought in South Korea were reported to be authentic. In most cases, labeled *Panax* species were substituted with other *Panax* species, but substitution of ginseng root, the medicinally recommended plant part, with leaves, stems or flowers was also reported. Efficient and practical authentication using biomarkers to distinguish the main ginseng varieties and secondary metabolite spectra for age determination are essential to combat adulteration in the global marketplace.

## Introduction

Ginseng is used collectively to refer to several plant species, mainly in the Araliaceae genera *Panax* L. and *Eleutherococcus* Maxim. In China, the ginseng preparations have been used for thousands of years in traditional medicine (Robbins, 1998). Demand for ginseng roots in the 18th century intensified the wild harvest of the main species *Panax ginseng* C. A. Mey. (*Araliaceae*) (Korean ginseng), and nearly extirpated it from the wild (Millspaugh, 1974), but also fuelled a rapid expansion in wild-harvesting of *Panax quinquefolius* L. (*Araliaceae*) (American ginseng) that in turn destroyed wild populations in North America (Kimmens, 1975). There are currently 13 recognized ginseng species, but new taxa at species, subspecies and variety level are continuously published (Manzanilla et al., 2018). The most commonly used species in the genus are *P. ginseng*, *P. quinquefolius*, *Panax notoginseng* (Burkill) F. H. Chen (*Araliaceae*) (Chinese ginseng), *Panax japonicus* (T. Nees) C. A. Mey. (*Araliaceae*) (Japanese ginseng), *Panax pseudoginseng* Wall. (*Araliaceae*) (Himalayan ginseng) and *Panax vietnamensis* Ha and Grushv. (*Araliaceae*) (Vietnamese ginseng) (Yang and Wu, 2016). The majority of commercialized ginseng material is from cultivation and controlled sustainable wild harvest, whereas material from uncontrolled depletive wild harvesting plays a minor and decreasing role. *P. ginseng, P. quinquefolius* and *P. notoginseng* are the three most widely used species in herbal medicine, and are traded as either red ginseng (Ginseng Radix et Rhizoma Rubra), white ginseng (Ginseng Radix et Rhizoma), American ginseng (Panacis Quinquefolii Radix), or notoginseng (Notoginseng Radix et Rhizoma). These ginseng varieties are sold in different stages of processing from raw materials through highly processed products that have lost their botanical morphological characteristics to extracts differentiated mainly by ginsenoside content (Yang and Wu, 2016). In the global market, many different formulations of these herbs are available, including gelcaps, capsules, teas, tinctures, slices to eat in salads, powders, as well as entire roots. There are a wide variety of products that contain ginseng such as toothpaste, cigarettes, soaps, cosmetics, beverages (including beer), coffee, baby food, candies, and gum (Morgan and Cupp, 2010).

In the global medicinal plant trade, the economic value of ginseng is estimated to be more than US$ 2.1 billion (Manzanilla et al., 2018). Prices vary widely based on the quality and the quantity of the ginseng root. The ginseng cultivar, locality, growth condition (cultivated or harvested from the wild), and plant age are some of the most important factors which influence the quality and properties of *P. ginseng* (Kim et al., 2012). It was shown previously that *P. ginseng* possesses different active ingredients and differentiated curative effects depending on the age of the plants, that is why the older ginseng plants are sold at a much higher price than the younger ones (Kim et al., 2011; Yang et al., 2012; Pan et al., 2013). Tenfold and more price differences among the main *Panax* processed varieties is the main driver for intentional adulteration (Choi et al., 2007). Adulteration using low-price varieties, low grade material from above-ground parts or processing waste, alternative species, or nothing at all, could bring huge profits through deceptive or illegal trade (Huang et al., 2017). The authentic herbal medicines and food supplements should be non-adulterated (Simmler et al., 2018) while the inadvertent or intentional adulteration (Simmler et al., 2018) includes contamination, product substitution and the use of fillers (Shanmughanandhan et al., 2016).

Efficient discrimination methods for ginseng varieties is necessary to combat fraud. Product adulteration and substitution are severe and widespread problems in the ginseng market, given the significant difference in medicinal value and economic benefits of different ginseng preparations (Zhao et al., 2020). The adulteration interferes with the proper and correct use of ginseng products and compromises the credibility of the whole supply chain. The development of efficient and practical authentication using biomarkers to distinguish the main ginseng varieties and secondary metabolite spectra for age determination are essential to combat adulteration. This review aims to provide an overview of the state of the art in ginseng authentication, while also highlighting current strengths and limitations.

## Methods

### Search Strategy

Four databases (Web of Science, PubMed, Scopus, and ScienceDirect) were systematically searched for peer reviewed articles using keywords combined with Boolean operators: [(ginseng OR Panax) AND (identification OR authentication OR authenticity OR authentic OR adulteration OR contamination OR substitution)] following the PRISMA guidelines (Moher et al., 2009). After the search was performed (on 17 February 2020), weekly updates were received and taken into consideration as the option “search alert” has been activated for all four literature databases.

### Selection Process and Criteria

A total of 3,683 records were identified, with WoS = 1,277, PubMed = 692, Scopus = 1,480, ScienceDirect = 234 respectively, as well as another 49 records from other sources. Out of these a total 1,023 records were retained after removing duplicates. After screening, 869 records were excluded. The highly diverse reporting formats of the authenticity results made it necessary to define unifying criteria for selection and retain only the relevant articles in our review. The remaining 154 full-text articles were assessed for eligibility using the assessment criteria: 1) The reported samples had to be “herbal products” in the broad sense. The widest possible range of commercial names was used for the searches and accepted for inclusion in the review; 2) The analyzed products had to be “commercial.” The “cost-free,” “gifts” or the “donated” products were excluded from our analysis; 3) The products had to be allocated specifically to a “country” or “territory” (i.e., Hong Kong, European Union); 4) The “authentic” or “adulterated” conclusion was drawn by the authors of the reviewed articles; 5) All authentication methods were accepted. After applying these selection criteria, 120 articles were excluded because they did not report authentication results of commercial herbal products. This objective literature search identified 34 peer-reviewed publications reporting authentication of a total of 507 ginseng-containing commercial herbal products (Table 1). Authentication of botanical identity was reported for 468 products in 29 studies, and 13 of them used only DNA-based methods, mostly DNA barcoding. In another 13 studies only chemical methods were used, while the remainder used a combination of genetic and chemical authentication methods. Fifty-seven percent of studies (17 out of 30) reported authentication results for ginseng products purchased from China. The integrity of the chemical composition of 39 ginseng products was reported in five additional peer-reviewed studies.

**TABLE 1:** The authenticity of ginseng commercial herbal products sold on the global market.

Country/territory	Labeled and authenticated Panax sp./type of herbal product	Identified adulterant	Product composition	Products			Authentication methods	Ref
				Total	Authentic	Adulterated		
				no	no	no		
	*Botanical identity*							
Brazil	*P. ginseng*	*Pfaffia* spp.	Root products (0.2% ginsenoside Rg1 and 0.1% ginsenoside Rb1, HPLC)	5	0	5	DNA barcoding	(Palhares et al., 2015)
China	*P. ginseng, P. quinquefolius, P. notoginseng*	n/a	Batches of TCM compound preparations (e.g. pill, bag, injection, capsule, tablet, powder, dripping pill)	40	38	2	LC–MS	(Yang et al., 2016)
China	*P. ginseng*	*P. quinquefolius*	Batches of CPMs containing ginseng products	24	19	5	DNA barcoding	(Liu et al., 2016)
China	*P. ginseng*	*P. quinquefolius*	Ginseng Radix et Rhizoma samples	15	12	3	DNA barcoding	(Han et al., 2016)
China	*P. notoginseng*	n/a	Baches of CPM Shuxiong tablets prepared from Notoginseng Radix et Rhizoma), Carthami Flos, and Chuanxiong Rhizoma	12	12	0	UPLC/QTOF-Fast DDA	(Yao et al., 2016)
China	*P. ginseng, P. quinquefolius*	n/a	CPMs containing ginseng or American ginseng	11	8	3	MAS-PCR	(Cheng et al., 2015)
China	*P. ginseng, P. quinquefolius, P. notoginseng*	n/a	TCHMs multi-ingredient ginseng preparations, Sheng Mai Yin (SMY) capsule and granules	11	6	5	HPTLC	(Xie et al., 2006)
China	*P. notoginseng*	flower of *P. notoginseng*	TCM preparations with P. notoginseng powder (dry roots and rootstock)	10	9	1	UPLC/Qtof MS	(Liu et al., 2015)
China	*P. ginseng*	n/a	Ginseng containing samples	10	8	2	PCR	(Zhou et al., 2016)
China	*P. ginseng*	*P. quinquefolius, Platycodon grandiflorum* (Jacq.) A.DC. (*Campanulaceae*), *Physochlaina infundibularis*Kuang (*Solanaceae*), *Phytolacca acinosa* Roxb. (*Phytolaccaceae*)	Root samples	8	5	3	NIR barcode	(Dong et al., 2020)
China	*P. ginseng*	n/a	Ginseng Radix et Rhizoma (dried roots and rhizomes of *P. ginseng*)	3	3	0	DNA barcoding	(Zhang et al., 2019)
China	*P. ginseng, P. notoginseng*	n/a	steamed roots of *P. ginseng*; powdered roots of *P. notoginseng*	2	2	0	DNA barcoding	(Dong et al., 2014)
China	*P. ginseng*	n/a	Shihu Yeguang Pills containing Ginseng Radix Et Rhizoma	1	1	0	DNA barcoding	(Jiang et al., 2019)
China	*P. ginseng*	n/a	Batches of Asian ginseng	11	11	0	UPLC/Q-TOF-MS	(Li et al., 2010)
China	*P. quinquefolius*	n/a	Batches of American ginseng	7	7	0		
Canada	*P. quinquefolius*	n/a	Batches of American ginseng	5	5	0		
United States	*P. quinquefolius*	n/a	Batches of American ginseng	4	4	0		
China	*P. quinquefolius*	*P. ginseng*	Batches of American ginseng preparations	13	0	13		
China	*P. ginseng*	leaf/stem	Root extract	1	0	1	HPTLC, HPLC	(Govindaraghavan, 2017)
European Union	*P. ginseng*	*P. ginseng* leaf or other plant parts, *P. quinquefolius* roots	Root extracts, stem/leaf extract, berry extract	12	6	6		
Australia	*P. ginseng*	*P. ginseng* leaf or other plant parts	Capsules (extract or herb), tablet	4	1	3		
South Korea	*P. ginseng*	n/a	*P. ginseng* extract, tea, capsule	3	3	0	Multiplex PCR	(Tian et al., 2020)
China	*P. ginseng, P. quinquefolius*	n/a	*P. ginseng* extract, tea, capsule; *P. quinquefolius* extract, tea, capsule	6	6	0		
United States	*P. ginseng, P. quinquefolius*	n/a	*P. quinquefolius* extract, tea, capsule	3	3	0		
Hong Kong	*P. ginseng*	n/a	Ready-to-serve ginseng soup	1	1	0	Multiplex PCR, DNA sequencing	(Lo et al., 2015)
Hong Kong	*P. ginseng, P. quinquefolius*	n/a	Dried roots, powders, tea granules	7	7	0	PCR, HAD	(Jiang et al., 2014)
Italy	*P. ginseng, P. quinquefolius*	n/a	Raw materials (as body root, root tails and root prongs), capsules and tablets (containing dried extract)	19	12	7	RFLP, HPLC	(Del Serrone et al., 2006)
South Korea	*P. ginseng*	n/a	decoctions, beverages, capsules, tablets	61	61	0	HPLC, UPLC–DAD–ESI-IT-TOF-MS	(Choi et al., 2018)
South Korea	*P. ginseng*	n/a	Bak-Ho-Ga-Insam-Tang resources containing *P. ginseng*	14	14	0	Gradient PCR	(Shim et al., 2005)
Taiwan	*P. ginseng*	not composed of 6 years old ginseng radix only	White ginseng radix sliced material, powder, capsules	7	6	1	1H-NMR	(Lin et al., 2010)
China	*P. ginseng*	n/a	white ginseng radix sliced material	1	1	0		
Taiwan	*P. ginseng*	Panacis quinquefolii Radix	Chinese medical preparations containing Ginseng Radix	58	27	31	nested PCR, RFLP, DNA sequencing	(Lu et al., 2010)
United Kingdom	*P. ginseng, P. quinquefolius, P. notoginseng*	n/a	American ginseng, white Asian ginseng, sanchi ginseng	8	8	0	LC-MS	(Kite et al., 2003)
United States	*P. ginseng*	*Astragalus propinquus* Schischkin (*Leguminosae*)	Single ingredient HMP	1	0	1	DNA barcoding	(Molina et al., 2018)
United States	*P. ginseng, P. quinquefolius*	n/a	Ground *P. quinquefoliu*s root (capsule), red *P. ginseng* root extract (liquid vial)	2	2	0	UPLC/QTOF-MS	(Yuk et al., 2016)
United States	*P. quinquefolius*	*P. ginseng*	American ginseng products	6	4	2	HPLC	(Yu et al., 2014)
United States	*P. ginseng, P. quinquefolius*	soybean	American and Korean ginseng fresh or dried roots, powders, capsules, tablets	24	22	2	PCR, RAPD, HPLC	(Mihalov et al., 2000)
China	*P. ginseng, P. quinquefolius*	n/a	American and Korean ginseng dried root	2	2	0		
United States and Canada	*P. ginseng, P. quinquefolius*	*P. quinquefolius*	NHPs containing Red Korean, Korean, American, sand ginseng (capsules, tablets, roots, carved roots, extracts, teas, and dried and shredded products	36	22	14	DNA barcoding	(Wallace et al., 2012)
Total				***468***	***358***	***110***		
	*Chemical composition*							
China	Functional food for relieving physical fatigue	Testosterone, adalafil, sildenafil	Fur seal ginseng pills with complex herbal composition, including *P. ginseng*	16	13	3	HPLC	(Wang et al., 2019)
China	Antidiabetic functional food	Tolbutamide, glimepiride, metformin	Bitter melon and ginseng soft gels containing also containing American ginseng root	16	13	3	UPLC-Q-Orbitrap-MS/MS	(Xie et al., 2019)
China	Herbal medicines for male sexual health	Sildenafil, hongdenafil, vardenafil, homosildenafil	Complex herbal composition, including Radix et rhizoma ginseng or Radix et rhizoma ginseng rubra (tablet, capsule, pills, soft gel capsule)	4	0	4	TLC, HPLC-MS	(Cai et al., 2010)
Saudi Arabia	Dietary supplements for weight loss, slimming and as a stimulant or stamina enhancer	Theobromine, theophylline, pseudoephedrine, caffeine, hydrochlorothiazide, yohimbine	Ginseng extract (capsule), Korean Ginseng (capsule)	2	2	0	UHPLC–DAD	(Ahmad et al., 2020)
Sweden	Dietary supplement	Ephedrine	Ginseng preparation	1	0	1	n/a	(Cui et al., 1994)
***Total***				***39***	***28***	***11***		
**TOTAL**				**507**	**386**	**121**		

## Results

The analysis of the botanical and chemical identity of 507 ginseng-containing commercial herbal products shows that 76% (*n* = 386) are authentic while 24% (*n* = 121) were reported as adulterated. These ginseng commercial herbal products were purchased from 12 countries scattered across six continents: Asia (*n* = 375), North America (*n* = 81), Europe (*n* = 40), South America (*n* = 5), Australia (*n* = 4), and Africa (*n* = 2). Among continents, the number of commercial products as well as the percentage of adulteration varies significantly, being the highest in South America (100%, *n* = 5) and Australia (75%, *n* = 3), and lower in Europe (35%, *n* = 14), North America (23%, *n* = 19), Asia (21%, *n* = 80) and Africa (0%) (Table 1).

Analysis of authentication results per country (*n* = 459 commercial ginseng products were clearly allocated to a single country) show that the number of samples purchased and tested for each country varies. In China, 224 ginseng products were successfully authenticated, representing almost half (49%) of all reported samples worldwide, while South Korea (*n* = 78), Taiwan (*n* = 65) and United States (*n* = 40) follow distantly. All of these countries, except Taiwan, are the three major ginseng cultivating countries in the world. This data also suggests the importance of these herbal products in Asian traditional medicine systems is mirrored by the interest of the scientific community to develop and test new methods on market products. Much smaller numbers of tested commercial ginseng products were reported for Italy (*n* = 19), Hong Kong (*n* = 8), United Kingdom (*n* = 8), Brazil (*n* = 5), Canada (*n* = 5), Australia (*n* = 4), Saudi Arabia (*n* = 2), and Sweden (*n* = 1), but they support and reconfirm the widespread interest for ginseng-containing products on the global market.

The ginseng authenticity reported for the 12 countries represented in our review range widely from country to country. The five countries for which more than 10 products have been successfully authenticated account together for 84% of all ginseng products analyzed worldwide. Out of these countries, the highest percentage of adulterated ginseng products were purchased from Taiwan (49%), followed by Italy (37%), China (21%), United States (12%), while all the products bought from the South Korean market were reported to be authentic. Notably, six out of the seven remaining countries, each with less than 10 authenticated commercial samples, have all their products reported either as authentic (i.e., Canada, Hong Kong, Saudi Arabia, United Kingdom), or adulterated (i.e., Brazil, Sweden) while the ginseng products purchased from Australia were reported to be 25% authentic and 75% adulterated (Table 2).

**TABLE 2:** National distribution of the ginseng commercial ginseng herbal products and their authenticity.

Country	Products (total)	Authentic/adulterated			
	no	no	%*	no	%*
China	224	176	79%	48	21%
South Korea	78	78	100%	0	0%
Taiwan	65	33	51%	32	49%
United States	40	35	88%	5	12%
Italy	19	12	63%	7	37%
Hong Kong	8	8	100%	0	0%
United Kingdom	8	8	100%	0	0%
Brazil	5	0	0%	5	100%
Canada	5	5	100%	0	0%
Australia	4	1	25%	3	75%
Saudi Arabia	2	2	100%	0	0%
Sweden	1	0	0%	1	100%

* The percentage values were rounded to the nearest whole number.

## Discussion

### Authenticity of Commercial Ginseng Herbal Products Sold on the Global Market

Overall, the peer-reviewed publications show that one in each four ginseng containing herbal products sold on the market is adulterated with respect to the labeled species or declared chemical composition. Adulterated ginseng products are present across many regions and countries, and this confirms a recent global analysis of DNA-based authenticity testing, both in terms of adulteration percentage and geographic spread (Ichim, 2019). In addition, our analysis further confirms the widespread presence of adulterated commercial herbal products in traditional medicine systems, including Traditional Chinese Medicine (TCM) (Han et al., 2016) and Ayurvedic medicine (Seethapathy et al., 2019).

In most cases, labeled *Panax* species are substituted with other *Panax* species. *P. ginseng* is replaced by *P. quinquefolius* in China (Han et al., 2016; Liu et al., 2016; Dong et al., 2020) and Taiwan (Lu et al., 2010), and vice-versa in United States (Li et al., 2010; Yu et al., 2014), so that the species most highly valued in that country is substituted by other ginseng species. The adulteration of traditional ginseng medicinal products included the substitution of ginseng root, the medicinally recommended plant part, with leaves, stems (Govindaraghavan, 2017) or even flowers (Liu et al., 2015), all replacing the high value herbal material with less costly ones. Liu et al. (2017) showed that ginsenoside ratios could be used to determine the age of cultivated *Panax ginseng* with 30-fold differences in some ginsenosides with age. Premium ginseng commercial products sold on the Taiwanese market have been reported to be adulterated with inferior plant material (Lin et al., 2010). Economically motivated adulteration of commercial ginseng products includes the use of soybean as the only plant ingredient identified in commercial products sold in the United States (Mihalov et al., 2000) thus confirming the reported widespread adulteration of commercial herbal products on the North American market (Newmaster et al., 2013).

The widespread use of the ginseng vernacular name across many countries and continents, including for species not from the same genus or even family (Osathanunkul and Madesis, 2019), is a catalyst conducive to adulteration. In Brazil for example, ginseng products have been found to be adulterated with a completely unrelated species from another family, *Pfaffia* spp., albeit locally known as Brazilian ginseng (Palhares et al., 2015). The inadvertent contamination through plant misidentification during harvesting or cross-contamination during processing but also the intentional and fraudulent use of filler species or cheaper substitutes, present on the herbal product market (Jordan et al., 2010; Sgamma et al., 2017; Ichim et al., 2020), also affects the highly valued ginseng-containing food supplements and traditional medicines across the globalized market. The presence of substitutes or filler species in some cases reflects intentional, economically motivated and fraudulent practices by producers or vendors although the European Pharmacopoeia (Ph. Eur.) and the United States Pharmacopeia (USP) as well as some monographs for herbal raw materials, allow a certain amount (e.g., 2% in USP) of foreign organic matter (Parveen et al., 2016; Sgamma et al., 2017) as acceptable accidental contamination. Counterfeiting by adding synthetic prescription drugs as chemical adulterants is another form of falsification, and Calahan et al. (2016) found that 28% of authenticated ginseng products in their study were adulterated. Apart from the many cases of adulteration and general low quality of commercial herbal products, *P. ginsen*g was also reported, along with some other plant species, as adulterant of a herbal product sold in Australia, supposedly to contain only *Eucalyptus radiata* A. Cunn. ex DC. (*Myrtaceae*) and *Melaleuca alternifolia* (Maiden and Betche) Cheel (*Myrtaceae*). This latter case suggests either accidental contamination through poor manufacturing process or intentional adulteration for achieving an expected physiological or pharmacological effect (Hoban et al., 2020).

### Ginseng-Drug Interactions, Abuse and Negative Effects of Long-Term Use on Human Health

The presence of unlabeled species, plant extracts or synthetic chemical compounds might negatively interact with other medicinal plants, food supplements or prescription drugs and will pose significant risks for human health (Jordan et al., 2010). Adverse drug reactions (ADRs) due to herb–drug interactions (HDI) can appear in patients taking herbs and prescribed medications concomitantly (Awortwe et al., 2018). Ginseng can interfere with various drugs, such as digoxin, insulin, anticoagulants, and monoamine oxidase inhibitors (Sellami et al., 2018). Pharmacovigilance relies heavily on ADR reporting, and despite initiatives to stimulate reporting of suspected ADRs associated with herbal medicines, numbers of herbal ADR reports are relatively low. ADRs Under-reporting is likely to be specific for herbal medicines, since their users usually do not look for medical advice about their use of such products, or report if they experience any adverse effects (Barnes, 2003).

The specific active constituents in Panax herbs, the ginsenosides, have been shown to improve immune function, reduce mental stress, and stabilize blood pressure while ginseng products are used as an endurance performance enhancer (Sellami et al., 2018). Nevertheless, authentic ginseng products does not represent a doping concern for athletes, as there were no positive tests for any International Olympic Committee (IOC) banned or restricted substances in any of the subjects after the ingestion of commercially available, proprietary ginseng root extract product (Goel et al., 2004).

Adverse drug reactions to ginseng are associated with high doses and long-term usage (Kiefer and Pantuso, 2003; Sellami et al., 2018). The ginseng abuse syndrome includes edema, decreased appetite, depression, and hypotension (∼10%), hypertension (17%), sleeplessness (20%), nervousness (25%), skin eruption (25%), and morning diarrhea (35%) (Paik and Lee, 2015). Long term use may cause blood clotting (Mohammed Abdul et al., 2018). Ginseng reduces the blood levels of warfarin and alcohol as well as induced mania if taken concomitantly with phenelzine, a non-selective and irreversible monoamine oxidase inhibitor used as an antidepressant and anxiolytic agent (Chen et al., 2011). Because the ginsenosides have a chemical structure similar to that of testosterone, estrogen, and glucocorticoids, the ginseng may also produce effects similar to those of estrogen (Anadón et al., 2016). Moreover, women may experience additional side effects, such as vaginal bleeding and breast tenderness. Most of these side effects are serious enough to warrant stopping taking ginseng in breast cancer patients (Sellami et al., 2018). A rare adverse drug reaction to herbal and dietary supplementation, the drug-induced liver injury (DILI), was reported as result from ingestion of ginseng for premenopausal symptoms (Lin et al., 2018). Recently, a website with a critically reviewed database presenting reported cases of ginseng-drug interactions was publicly launched (Wu et al., 2019).

The herbal medicines differ considerably from conventional medicines, and they pose a variety of challenges to their pharmacovigilance. For herbal pharmacovigilance, four main challenges: 1) Substitution and adulteration; 2) Nomenclature of herbals and ingredients of plant origin; 3) Lack of monitoring; and 4) Standardization, have been identified (de Boer et al., 2015). This review highlights that all four apply to ginseng commercial products, and support the challenge of detecting significant adverse drug reactions in a timely manner to protect consumers.

## Conclusion

Despite being the most valuable herbal product in terms of market value share, ginseng products are poorly regulated. Several authentication studies have shown that adulteration is not uncommon and not limited to any specific country of origin. The high market value of ginseng provides an incentive for fraudulent actors to generate profits at the expense of gullible consumers and honest producers, wholesalers and retailers. Although authentication using traditional, pharmacopoeial, analytical methods such as TLC, HPLC and NMR can be used for advanced quality control, the standard authentication protocols are insufficient for efficiently detecting species adulteration, adulteration with synthetic pharmaceuticals and spiking of low quality products with marker compounds. Cutting-edge approaches enable distinction of age-specific metabolite spectra, quantification of active ingredients and accurate identification of ginseng species, but these are not yet in widespread use.

## Author Contributions

MI performed the literature systematic search and analyzed the results. MI and HB wrote the manuscript together.

## Funding

This publication was supported by the National Core Program funded by the Romanian Ministry of Research and Innovation, project number 25N/11.02.2019, BIODIVERS 19270401 (for MI).

## Conflict of Interest

The authors declare that the research was conducted in the absence of any commercial or financial relationships that could be construed as a potential conflict of interest.
